# Regulation of hepatitis C virus replication via threonine phosphorylation of the NS5A protein

**DOI:** 10.1099/jgv.0.000975

**Published:** 2017-11-15

**Authors:** Niluka Goonawardane, Douglas Ross-Thriepland, Mark Harris

**Affiliations:** School of Molecular and Cellular Biology, Faculty of Biological Sciences and Astbury Centre for Structural Molecular Biology, University of Leeds, Leeds, LS2 9JT, UK; ^†^​ Present address: AstraZeneca, Cambridge Biomedical Campus, Cambridge, CB20AA, UK.

**Keywords:** hepatitis C virus, NS5A, phosphorylation, RNA replication, infectivity, Huh7 cells

## Abstract

The hepatitis C virus non-structural 5A (NS5A) protein is highly phosphorylated and plays roles in both virus genome replication and assembly of infectious virus particles. NS5A comprises three domains separated by low complexity sequences (LCS). Mass spectrometry analysis of NS5A revealed the existence of a singly phosphorylated tryptic peptide corresponding to the end of LCS I and the beginning of domain II that contained a number of potential phosphorylatable residues (serines and threonines). Here we use a mutagenic approach to investigate the potential role of three of these threonine residues. Phosphomimetic mutations of two of these (T242E and T244E) resulted in significant reductions in virus genome replication and the production of infectious virus, suggesting that the phosphorylation of these residues negatively regulated virus RNA synthesis. Mutation of T245 had no effect, however when T245E was combined with the other two phosphomimetic mutations (TripleE) the inhibitory effect on replication was less pronounced. Effects of the mutations on the ratio of basally/hyperphosphorylated NS5A, together with the apparent molecular weight of the basally phosphorylated species were also observed. Lastly, two of the mutations (T245A and TripleE) resulted in a perinuclear restricted localization of NS5A. These data add further complexity to NS5A phosphorylation and suggest that this analysis be extended outwith the serine-rich cluster within LCS I.

## Introduction

Hepatitis C virus (HCV) is a globally prevalent human pathogen and a leading cause of liver cirrhosis and hepatocellular carcinoma, accounting for up to 500 000 related deaths per year [1]. Although the residual risk remains largely unknown, recent development of potent direct acting antivirals has allowed patients to be rapidly cured of HCV infection, with a concomitant reduction in related hepatic complications [2]. HCV is a member of the *Hepacivirus* genus in the family *Flaviviridae* [3]. HCV has a positive-strand RNA genome (∼9.6 kb in length) that encodes a large polyprotein (∼3000 amino acids) flanked by 5′ and 3′-UTRs. The polyprotein is processed by viral and host proteases into 10 viral proteins: core, envelope glycoproteins E1 and E2, the viroporin p7, and the non-structural (NS) proteins NS2-NS5B [4, 5]. The structural proteins, core, E1 and E2, are essential components of the HCV virion and necessary for viral assembly, entry and fusion [6, 7], whereas p7 and NS2 are required for assembly of, but do not form part of, infectious virus particles. NS3 to NS5B are necessary and sufficient to form a membrane-associated genome replication complex [7]. Numerous prior studies demonstrated that HCV (mediated in particular by NS4B and NS5A) induces extensive rearrangement of the endoplasmic reticulum to create a membranous web, which is essential for viral RNA replication [7–9].

**Fig. 1.**
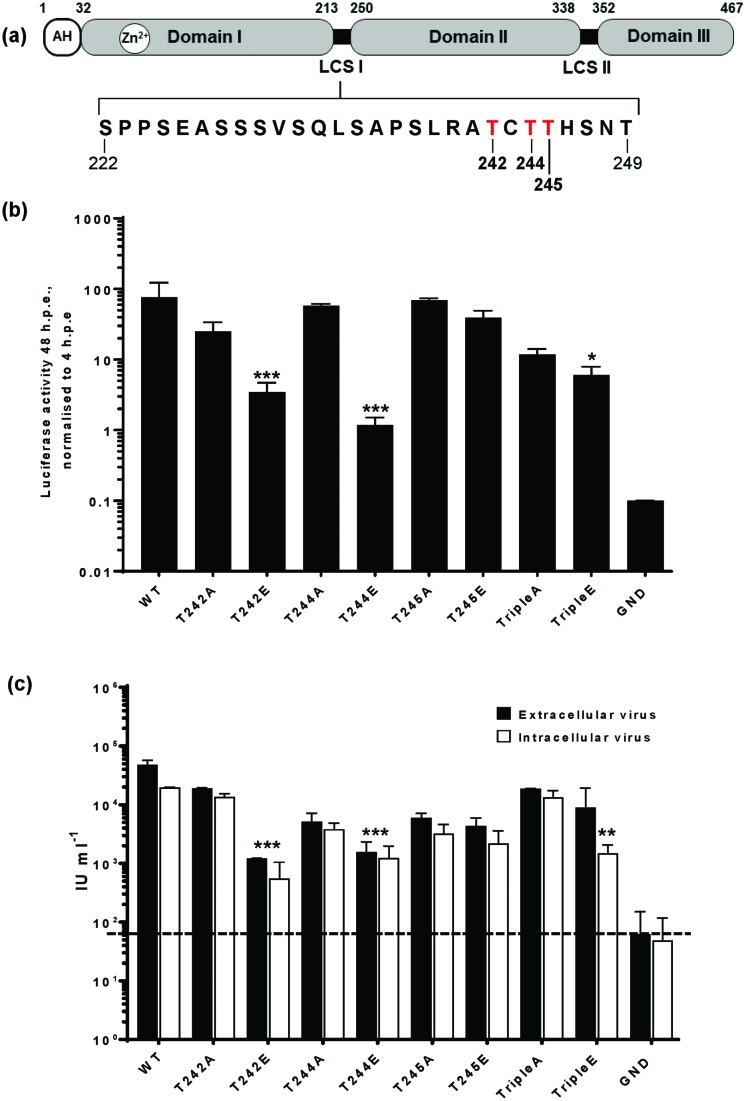
Mutational analysis of the role of NS5A threonines 242, 244 and 245 in the HCV lifecycle. (a) Schematic of NS5A structure showing amino acid residues for LCS I and location of the amphipathic helix (AH). The three threonine residues are highlighted in red. (b) Replication of threonine mutants in the context of the mSGR-luc-JFH-1. Huh7 cells were electroporated with *in vitro* transcripts of the indicated mSGR-luc-JFH-1 mutants. Luciferase activity was measured at 48 h post-electroporation (hpe) and was normalized to 4 hpe. (c) *In vitro* transcripts of mJFH-1 containing the indicated mutations were electroporated into Huh7 cells. Intracellular and extracellular infectious virus was titrated at 48 hpe by using the Incucyte-ZOOM as described [29]. Data from three independent experiments are shown and error bars represent the standard error of the mean. Significant differences are denoted by *P<0.05, **P<0.01 or ***P<0.001.

NS5A (~450 amino acid) is a multi-functional phosphoprotein that is anchored to the membranous web by an N-terminal amphipathic helix. Despite having no identified catalytic function, NS5A plays a key role in the HCV lifecycle, and is implicated in both genome replication and virus assembly. These functions are likely mediated by protein–protein interactions. It consists of three domains (I, II and III) that are separated by low complexity sequences (LCS); LCS I is serine-rich and LCS II is proline-rich (Fig. 1a) [10]. The structure of domain I has been resolved [11–13], revealing four different dimeric arrangements of the monomeric unit. Domain I of NS5A exhibits high sequence homology among all HCV genotypes whereas domains II and III exhibit a lower level of homology [14, 15].

NS5A exists in two phosphorylated forms, distinguished by their migration on SDS-PAGE. These are referred to as the basal (p56) and hyperphosphorylated (p58) species of NS5A [16–18]. It has been proposed that phosphorylation of NS5A may play a role in regulating its differential functions at different stages of the virus lifecycle, for example effecting a switch between genome replication and virus assembly. To assess the functions of NS5A phosphorylation, we and others have used mutational analysis and mass spectrometry to map the target sites of phosphorylation [19–23]. These studies have identified multiple phosphorylation sites within the LCS I that exhibit profound multi-functional effects on HCV lifecycle. In particular, serines (S) 222, 225 and 235 have been shown to regulate HCV genome replication [23–26], and in a recent study, we revealed the involvement of S225 in host protein interactions and control of the sub-cellular localization of NS5A [19, 20]. A number of phosphorylated threonines (T) have also been identified, mainly within the C-terminal region of NS5A. These include T348 and T360 [22, 24, 25], which are also likely to play a role in RNA replication. It should be noted that the effect of phosphorylation can vary between the genotypes, and the determinants for this apparent genotype-specific replication capacity are largely unknown. This can be exemplified by the previously identified S249 (S2221 in polyprotein numbering) in the genotype 1b Con1 isolate as an important phosphorylation site [26], however, mutational analysis of the corresponding T249 residue in JFH-1 resulted in no phenotype.

Here, we extend our functional analysis of phosphorylation sites within LCS I [27] to investigate the role of three previously uncharacterized threonine residues; 242, 244 and 245, in the context of both the JFH-1 cell culture infectious virus and a corresponding subgenomic replicon. By site-directed mutagenesis, we show here that the phosphorylation of T242 and T244 likely acts as a negative regulator of RNA replication. In contrast, T245 phosphorylation alone has no phenotype, but could partially compensate for mutations at T242/244. We show using confocal microscopy that ablating phosphorylation of T245 resulted in a perinuclear restriction of NS5A. T245 phosphorylation appeared to contribute to hyperphosphorylation as the T245A phosphoablatant mutation exhibited a reduction in levels of the hyperphosphorylated NS5A species, further illustrating the complexity of phosphorylation within the LCS I of NS5A.

## Results

### Phosphorylation of threonine 242 and 244 negatively regulates HCV genome replication

We previously used mass spectrometry to identify phosphorylated residues in JFH-1 NS5A [27]. As well as a number of phosphorylated serines, we identified a tryptic peptide corresponding to residues 241–273 that contained one phosphorylated residue, however we were unable to unambiguously identify the site of phosphorylation. As we had also previously demonstrated that S247 and T249 mutations had no phenotype in either SGR or infectious virus [26], we focussed on the three threonine residues at the N-terminus of the tryptic peptide – T242, 244 and 245 in LCS I. In order to understand the possible roles of these residues in the HCV lifecycle, we generated a series of point mutations to either alanine to block phosphorylation (phosphoablatant) or glutamic acid to mimic phosphorylation (phosphomimetic). As well as individual mutations, we mutated all three threonines either to alanine (TripleA), or glutamic acid (TripleE). These mutants were generated in the context of both mSGR-luc-JFH-1 and mJFH-1, constructs that had been engineered to contain unique restriction sites flanking NS5A to facilitate cloning and mutagenesis [28]. We firstly investigated the effects of these mutations on RNA replication. To do this *in vitro* transcribed mutant mSGR-luc-JFH-1 RNAs were electroporated into Huh7 cells, using an NS5B polymerase-inactive mutant (GND) as a negative control [29]. Luciferase activity 48 h post-electroporation (hpe) was normalized to the input assayed at 4 h as a measure of genome replication.

As shown in Fig. 1(b), none of the phosphoablatant mutations (T242A, T244A, T245A and TripleA) had any significant effect on replication, although T242A and TripleA exhibited a modest reduction that did not reach significance. These data suggest that the phosphorylation of these three threonine residues is not required for genome replication. In contrast, the phosphomimetic mutations T242E and T244E resulted in impaired replication, exhibiting up to 100-fold reduction compared to the wild-type. In contrast, T245E exhibited no significant defect in replication, resembling the parental mSGR-luc-JFH-1. Intriguingly the TripleE mutant had a more modest, yet still significant, phenotype, reducing replication by less than 10-fold. These results point to roles for T242, T244 and T245 in regulating HCV genome replication.

To investigate the potential effect of T242, 244 or 245 phosphorylation on production of infectious virus, *in vitro* transcripts of either wild-type or mutant full-length mJFH-1 virus RNA were electroporated into Huh7 cells. Both intracellular and extracellular infectivity was quantified by titration of either cell lysates or supernatants using the IncuCyte ZOOM as previously described [30]. As shown in Fig. 1(c), the results largely mirrored those seen for the SGR, all of the mutants were able to produce infectious virus, with the only significant reductions being observed for T242E, T244E and to a lesser extent the TripleE mutant. We conclude that phosphorylation of these threonine residues only affects genome replication, not virus assembly.

### Threonine phosphorylation regulates NS5A hyperphosphorylation

**Fig. 2.**
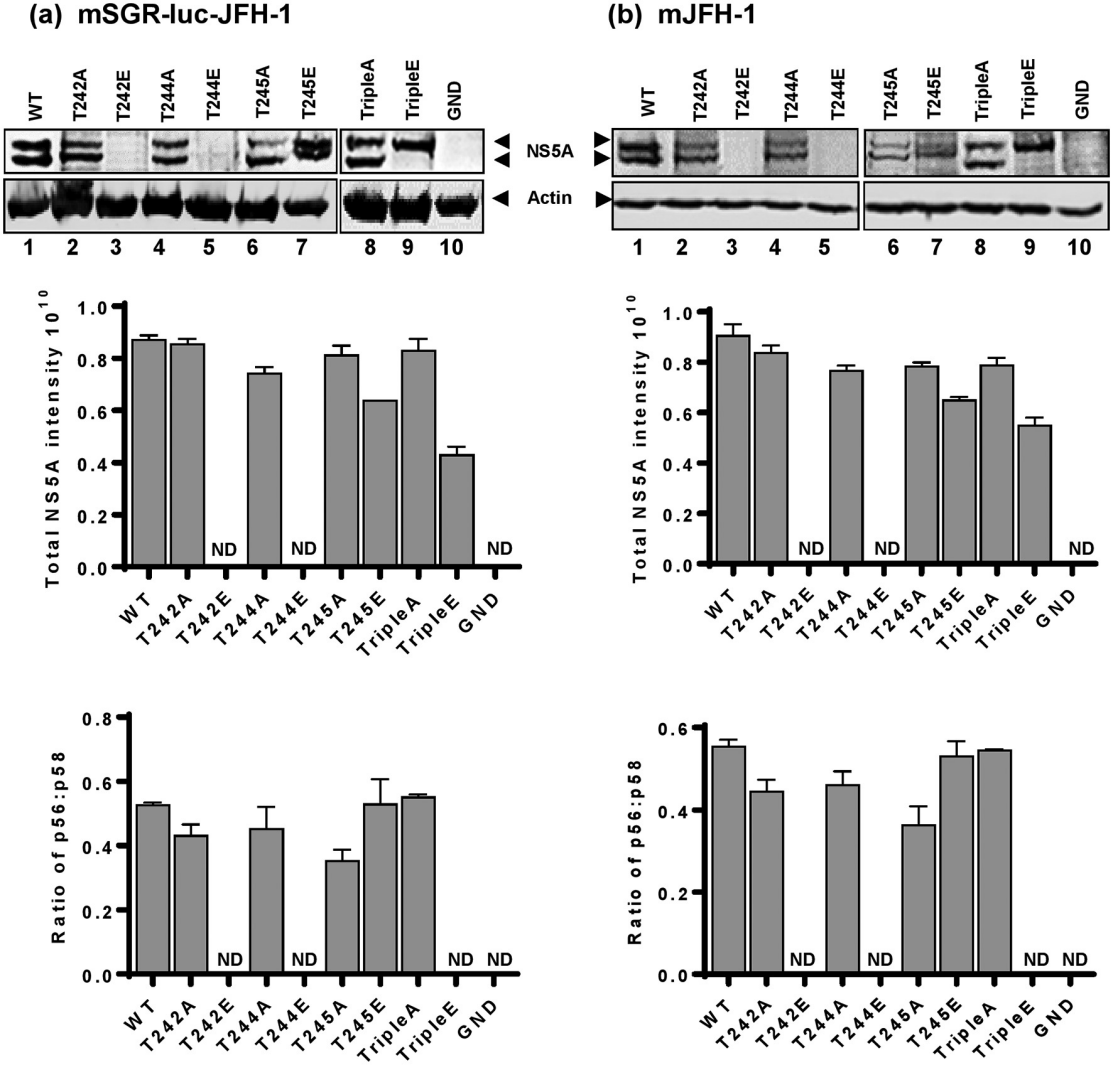
Analysis of NS5A protein expression for threonine mutants. Huh7 cells were electroporated with *in vitro* transcripts of either (a) mSGR-luc-JFH-1 or (b) mJFH-1 containing the indicated mutants, cells were lysed at 48 hpe and analysed by Western blot with sheep polyclonal anti-NS5A serum or anti-actin as the loading control. Western blots were quantified using Image Studio v3.1 (LI-COR). Data from three independent experiments are shown and error bars represent the standard error of the mean.

By Western blot analysis NS5A exhibits two distinct phosphorylated species, termed the hyper- and basal-phosphorylated forms. We [19, 20, 25, 27], and others [21, 22], have previously shown that hyperphosphorylation involves serine phosphorylation within LCS I, in particular serines 235 [23] and 225 [27], with evidence for hierarchical (or sequential) phosphorylation across LCS I [27]. We therefore asked whether these three threonines, that are located C-terminal to the LCS I serine cluster, might influence or regulate hyperphosphorylation. As shown in Fig. 2, we were able to detect NS5A by Western blot for all mutants apart from T242E and T244E, most likely due to the low levels of replication exhibited by these mutants. The single alanine mutations resulted in a subtle change in the migration of the basally phosphorylated form, this was more evident for TripleA where it can clearly be seen that the basally phosphorylated form migrated faster than the corresponding wild-type (Fig. 2a, b, compare lanes 1 to 8). The converse was observed for the phosphomimetic mutations: for T245E the basally phosphorylated form migrated slower than wild-type, and for TripleE the two forms co-migrated and could not be distinguished. These observations are consistent with a role for phosphorylation of these threonine residues in driving the hierarchical phosphorylation of serines within LCS I. The implication is that the basally phosphorylated NS5A can be converted into the hyperphosphorylated form by hierarchical phosphorylation across LCS I, involving both threonines and serines and resulting in an incremental decrease in mobility on SDS-PAGE. Quantitative analysis revealed only subtle differences in the ratio of basal:hyperphosphorylated NS5A (Fig. 2).

### Threonine phosphorylation may modulate the sub-cellular localization of HCV replication complexes

**Fig. 3.**
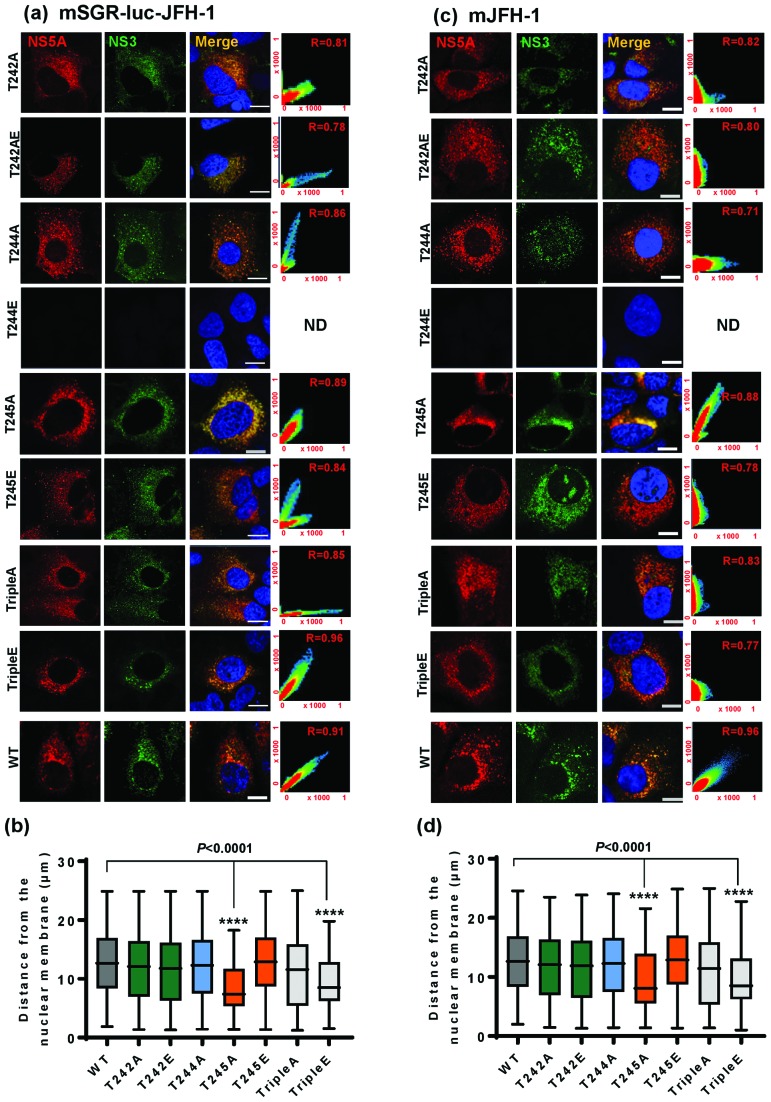
Analysis of the sub-cellular localization of NS5A and NS3. Huh7 cells were electroporated with *in vitro* transcripts of (a) mSGR-luc-JFH-1 or (b) mJFH-1 wild-type (WT) or the indicated mutants, seeded onto coverslips and incubated for 48 h prior to fixation. Cells were permeabilized and immunostained for NS5A (red panels) and NS3 (green panels). Scale bar, 20 µm. The scatter plots show the co-localization of NS5A and NS3 (*x* and *y* axes, pixel numbers). The correlation value (*r*) is shown for each image. Spatial data for NS5A were determined from 20 cells for each mutant or wild-type using the Image J software package. These data were used to determine the distance of NS5A from the nuclear envelope. The data shown are representative of three independent experiments. Significant difference from the control value is denoted by ****P<0.0001.

**Fig. 4.**
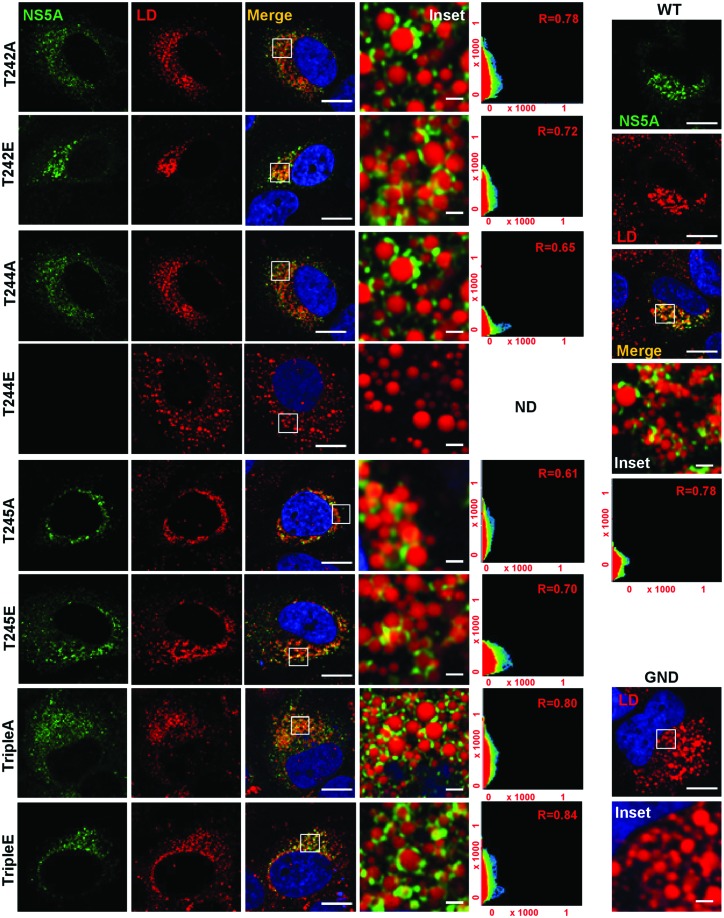
Analysis of the sub-cellular localization of NS5A and LD. Huh7 cells were electroporated with either wild-type (WT) or mutant mJFH-1 virus RNA, seeded onto coverslips and incubated for 48 h prior to fixation. Cells were permeabilized and immunostained for NS5A (green panels), and stained with Bodipy (558/568)-C12 dye to detect LD (red panels). The scatter plots show the co-localization of NS5A and LD (*x* and *y* axes, pixel numbers). The correlation value (*r*) is shown for each image. The nuclei were counterstained with DAPI (blue). Scale bars: 20 and 4 µm (insets).

**Fig. 5.**
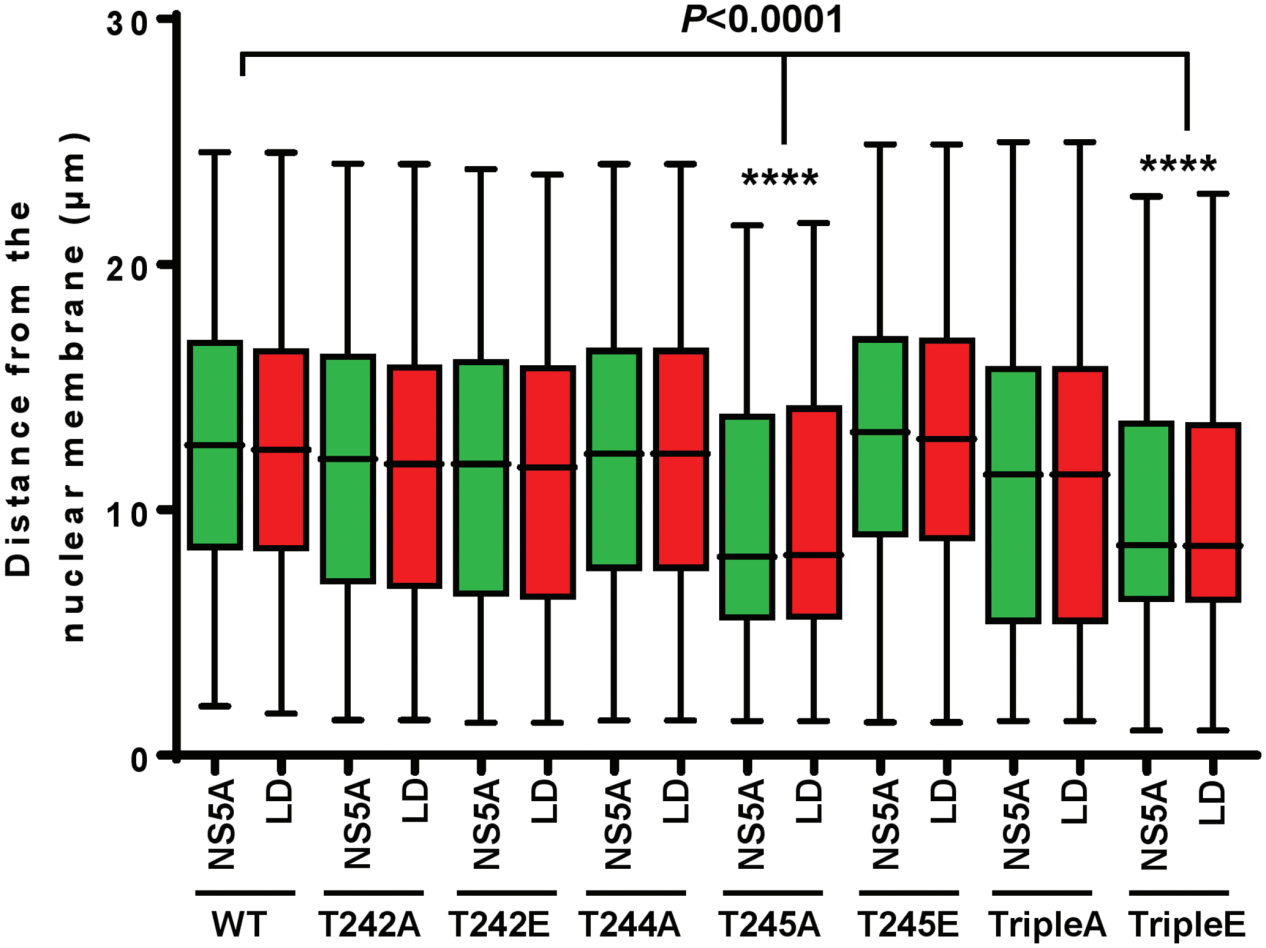
Analysis of NS5A and LD redistribution in infected cells. Spatial distribution of NS5A relative to LD in Huh7 cells electroporated with *in vitro* transcripts of either WT, or NS5A mutant mJFH-1 RNAs. Analysis was performed on the images shown in Fig. 4. Spatial data for NS5A were determined from 20 cells for each assay using the Image J software package. These data were used to determine the distance of NS5A and LD from the nuclear envelope. The data shown are representative of three independent experiments. Asterisks indicate significant difference from the control value ****P<0.0001.

We previously observed that phosphoablatant mutations in a subset of LCS I serines (notably S225), resulted in a perinuclear restricted distribution of NS5A and other replication complex factors (e.g. NS3), exemplified by a significant reduction in the distance of NS5A-positive punctae from the nuclear membrane [18]. We therefore sought to determine whether a similar phenotype was evident for the threonine mutants. Huh7 cells were electroporated with *in vitro* transcripts of either wild-type or mutant full-length mJFH-1 virus RNA and analysed by immunofluorescence (Fig. 3a, c) for both NS5A and NS3. As expected there was a strong co-localization of both viral proteins (scatter plots in right-hand images). Quantitative analysis was performed by measuring the distance of NS5A from the nuclear membrane in 20 cells (Fig. 3b, d). The majority of the mutants exhibited a wild-type distribution of NS5A and NS3, however two mutants (T245A and TripleE) exhibited the perinuclear restricted phenotype. This was evident in the context of both SGR (Fig. 3a, b) and infectious virus (Fig. 3c, d). As T245A replicated as wild-type (Fig. 1b, c) the perinuclear restriction was not due to reduced capacity for genome replication or NS5A abundance. Although T244E was able to replicate, albeit at low levels (Fig. 1b, c) we were unable to detect this NS5A mutant (or NS3) by immunofluorescence, this is consistent with the low expression levels (see Fig. 2). We also examined the distribution of NS5A in relation to cytoplasmic lipid droplets (LDs) (Fig. 4). For all of the mutants NS5A could be observed in discrete locations on the surface of LDs, with no obvious differences in the distribution between the various mutants and wild-type, suggesting that threonine phosphorylation does not influence the localization of NS5A to LDs. However, of note, LDs in cells infected with both T245A and TripleE exhibited the same perinuclear restriction observed for in Fig. 3. This was confirmed by spatial analysis (Fig. 5). Thus, we conclude that threonine phosphorylation is able to control the sub-cellular distribution of both replication complexes and LDs.

## Discussion

We present here the results of a mutagenic analysis of potential sites of threonine phosphorylation in LCS I of HCV NS5A. Our previous mass spectrometric analysis revealed the existence of a singly phosphorylated peptide corresponding to residues 241–273. This peptide corresponded to the C-terminus of LCS (residues 241–249) and a short segment of domain II, and contained five potential phosphorylated residues (T242, T244, T245, S247 and T249). As we had previously shown no phenotype for mutations of S247 and T249 [26] we focussed on the remaining three threonines. Of note, two of these, T242 and T244, are highly conserved in all genotypes of HCV, whereas T245 is conserved in all but genotype 4 where it is usually substituted by alanine. Our data reveal phenotypes in either replication or the sub-cellular localization of NS5A for mutations in all three residues – we conclude that all may be phosphorylation sites but with different functional outcomes.

Interestingly, phosphomimetic mutations of T242 and 244 significantly reduced genome replication whereas the corresponding phosphoablatant mutations had no effect. Paradoxically we also observed that mutation of T245 to either alanine or glutamate had no effect on genome replication, however when the T245E mutation was combined with T242E and T244E (TripleE) there was a partial restoration of replication. These data suggest that phosphorylation of T245 can override the inhibitory effect of phosphorylation at T242 or T244, however by itself it does not affect genome replication. A possible explanation is that T245 phosphorylation disrupts the recognition of T242 or T244 by cellular kinases.

These observations suggest that phosphorylation of T242 and/or T244 might negatively regulate HCV genome replication. The mechanism by which this might occur remains obscure, however one could speculate that phosphorylation of T242 or T244 could disrupt the interactions of NS5A with cellular protein(s) required for genome replication, or could recruit proteins that block this process. In this context it has been proposed that NS5A phosphorylation might drive a switch between genome replication and virus assembly – given that the positive-strand genome will need to function as a template for both processes. However, the phenotypes of mutants in phosphorylation sites within LCS I appear to be limited to effects on genome replication [26, 27]. Thus, although NS5A has been convincingly demonstrated to function at the level of infectious virus assembly, this is not regulated by LCS I phosphorylation. It is tempting to speculate that instead of the replication/assembly switch discussed above, LCS I phosphorylation might instead drive the switch between genome replication and translation. Both of these processes will require the genome as a template but are mutually exclusive as ribosomes and polymerases will move in opposite directions on the RNA template. Perhaps T242/T244 phosphorylation is involved in this switch?

In contrast to these data, phosphorylation of serines within LCS I has been shown to be required for genome replication. For example, the phosphomimetic S225D mutation had no effect on genome replication whereas the corresponding phosphoablatant S225A mutation reduced replication 10-fold [18, 26]. Taken together, these data thus point to a complex regulation of NS5A function by phosphorylation within LCS I – individual phosphorylation events can either negatively or positively influence genome replication.

Analysis of the distinct phosphorylated species of NS5A (basally and hyper-phosphorylated) by SDS-PAGE and Western blotting revealed a further interesting observation: for T245E and TripleE the apparent molecular weight of the basally phosphorylated species was increased. We previously analysed phosphomimetic mutants of serines in LCS I [19, 20] – our analysis revealed that the apparent molecular weight of the basally phosphorylated NS5A species increased as the position of the phosphomimetic residue was shifted towards the C-terminus, so, for example, the basally phosphorylated species of the S222D mutant was smaller than for S238D. Data presented here suggest that this trend continues into the potential phosphorylation sites C-terminal to S238. We interpret this observation to propose that T245 phosphorylation may prime a hierarchical phosphorylation cascade across LCS I. For the TripleE mutant both species of NS5A co-migrated, further implying that production of a hyperphosphorylated species may require complete phosphorylation of all three threonines together with an as yet undefined subset of serines within LCS I.

An additional level of complexity was observed when we analysed the sub-cellular localization of the different threonine mutants. Previously we had observed that for a subset of serine mutants (notably S225A); NS5A, together with other components of replication complexes (e.g. NS3 and PI4P lipids) were restricted to a perinuclear region. Although we do not yet understand the significance of this restriction, it was associated with a 10-fold reduction in replication, however it did not result from the reduced replicative capacity as other mutants with a similar defect showed a wild-type distribution [19, 20]. T245A and TripleE also showed this restriction, in the latter case this was associated with a 10-fold reduction in replication although T245A replicated as wild-type. These data suggest that the perinuclear restriction of NS5A phospho-mutants may depend on multiple factors. In this context our proteomic analysis revealed that S225A did not interact with a number of cellular proteins including nucleosome assembly protein 1-like protein 1 (NAP1L1) and bridging integrator 1 (Bin1), furthermore when these two proteins were depleted by siRNA the distribution of wild-type NS5A was also restricted [20]. Future work will investigate whether threonine mutants also abrogate binding to these (and other) cellular proteins. In addition, it will be of interest to determine whether phosphorylation of these threonine residues has any effect on the well-characterized function of NS5A to bind to, and activate, phosphatidylinositol 4-kinase IIIα, leading to accumulation of phosphatidylinositol 4-phosphate lipids [31], a function shown to be important for genome replication.

In conclusion, this study adds to the complex and confusing picture of NS5A phosphorylation. Ultimately a full understanding of the mechanisms and consequences of these post-translational events will require a panel of phosphospecific antibodies. Such reagents are available for a subset of the serines within LCS I [21, 23, 27], and have been informative, however there is a pressing need for similar antisera to be generated for the threonines identified in this study.

## Methods

### Plasmids

DNA constructs used were derived from the full-length pJFH-1 virus [32] and the luciferase sub-genomic replicon (pSGR-luc-JFH-1) [19, 29]. The mSGR-luc-JFH-1 and mJFH-1 constructs used throughout the study contained unique restriction sites flanking the NS5A coding sequence [28]. Site-directed mutagenesis of NS5A was performed with a Q5 Site-Directed Mutagenesis Kit (NEB) and correct substitutions were confirmed by sequencing.

### Cell culture

Huh7 cells were maintained in Dulbecco’s modified Eagle’s medium (DMEM; Sigma-Aldrich) supplemented with 10 % foetal bovine serum (FBS), 100 IU penicillin ml^−1^, 100 µg streptomycin ml^−1^, and 1 % non-essential amino acids (NEAA) in a humidified incubator at 37 °C with 5 % CO_2_. For virus propagation, the medium was supplemented with 25 mM HEPES.

### Electroporation of replicon and virus RNA constructs

The preparation of *in vitro* transcripts and electroporations for both mSGR-luc-JFH-1 and mJFH-1 were conducted as described previously [25]. In brief, 4×10^6^ Huh7 cells in diethyl pyrocarbonate (DEPC)–PBS were electroporated with 3 µg of *in vitro* transcribed RNA using a square-wave protocol at 260 V for 25 ms. Subsequently, cells were resuspended in complete DMEM and seeded into either 96-well plates (*n*=6) at 2.5×10^4^ cells well^−1^, or six-well plates (*n*=2) at 4×10^5^ cells well^−1^.

For a luciferase-based replicon assay, cells were harvested with passive lysis buffer (Promega) at indicated time points of 4 and 48 hpe. Luciferase activity was determined from 96-well samples on a BMG plate reader by automated addition of 50 µl luciferase assay reagent (Promega) and total light emission was monitored.

### Virus titration

HCV infectivity was determined by titration on Huh7 cells in a 96-well format as follows. For intracellular infectivity, at 48 hpe cells were suspended in PBS, lysed by five repetitive freeze-thaw cycles and clarified by centrifugation (2800 ***g*** for 5 min). For extracellular infectivity, the corresponding cell supernatant was also clarified by centrifugation. Samples were serially diluted twofold in complete DMEM before addition of 100 µl to cells that had been seeded 8 h earlier at 8×10^3^ cells well^−1^ (final volume, 200 µl). The counting of infected cells was automated by acquisition and analysis using an IncuCyte ZOOM platform. The virus titre was determined by averaging the positive cell count from three or more adjacent counted wells that gave the corresponding titres as described previously [30].

### SDS-PAGE/Western blot

Cells were washed twice in PBS, lysed in 1x Glasgow lysis buffer (GLB – 1 % (v/v) Triton X-100, 120 mM KCl, 30 mM NaCl, 5 mM MgCl_2_, 10 % (v/v) glycerol, 10 mM PIPES-NaOH, pH 7.2 with protease and phosphatase inhibitors) and harvested by centrifugation (2800 ***g***, 10 min, 4 °C) before determining and normalizing protein concentration by BCA assay (Pierce). Following separation by SDS-PAGE, proteins were transferred to a polyvinylidene difluoride (PVDF) membrane and blocked in 50 % (v/v) Odyssey blocking (OB) buffer (LI-COR) in Tris-buffered saline (TBS). The membrane was incubated with primary antibodies overnight at 4 °C, followed by secondary antibodies for 2 h at room temperature, both prepared in 25 % OB buffer. Primary antibodies used were; anti-NS5A (sheep, prepared in-house, 1 : 4000) and α-Actin (mouse, Sigma, 1 : 10 000). Secondary antibodies were anti-sheep (800 nm) or anti-mouse (700 nm), used at 1 : 10  000 prior to imaging using a LI-COR Odyssey Sa infrared imaging system. Quantification of Western blots was carried out using Image Studio v3.1 (LI-COR) using a background subtraction method.

### Immunofluorescence and confocal microscopy

Slides for immunofluorescence were prepared as described previously [20]. In brief, cells were washed with PBS before fixation for 20 min in 4 % (w/v) PFA, cells were subsequently permeabilized in PBS+0.1 % (v/v) Triton X-100, and blocked with PBS-T, 5 % (w/v) BSA before immunostaining with primary antibody (sheep anti-NS5A; 1 : 2000 or mouse anti-NS3; 1 : 1000). Various fluorescently conjugated secondary antibodies were used at 1 : 500 (Life Technology). Nuclei were counterstained with DAPI. Lipid droplets were stained using the BODIPY(558/568)-C12 dye at 1 : 1000 (Life Technology). Confocal microscopy images were acquired on a Zeiss LSM880 upright microscope with Airyscan, post-acquisition analysis was conducted using Zen software (Zen version 2015 black edition 2.3, Zeiss) or Fiji (v1.49) software [33].

### Quantification of NS5A distribution

For the quantification of NS5A spatial arrangement, images were acquired with the same acquisition parameters, but with variable gain to ensure correct exposure. The two-dimensional coordinates of the centroids of LD were calculated using the Analyze Particles module of Fiji (ImageJ). The distance of each particle to the edge of the nucleus, visualized using DAPI stain, was looked up using a Euclidean distance map computed with the Distance Transform module of Fiji and exported as a list of distance measurements via the Analyze Particle function. Box and whisker plots of these distance measurements were constructed using GraphPad Prism and compared between samples using one-way ANOVA and Bonferroni-corrected post hoc *t*-tests.

### Statistical analysis

Data sets were analysed using Student's *t*-test assuming a two-tailed, unequal variance to determine the statistical difference from wild-type (WT). *n*=3 or greater throughout and *P<0.05, **P<0.01, ***P<0.001. ****P<0.0001.
